# Research and development of the sOLVe Tube™ dual lumen endobronchial tube: from concept to construct

**DOI:** 10.3389/fmedt.2023.1158154

**Published:** 2023-09-15

**Authors:** Patricia Nwajuaku, Igor Barjaktarevic, Nir Hoftman

**Affiliations:** ^1^ Department of Anesthesiology and Perioperative Medicine, University of California Los Angeles, Los Angeles, CA, United States; ^2^ Division of Pulmonary and Critical Care Medicine, University of California Los Angeles, Los Angeles, CA, United States

**Keywords:** double lumen tube (DLT), one lung ventilation, endobronchial intubation, airway equipment, lung isolation

## Abstract

**Introduction:**

Dual lumen endobronchial tubes (DLTs) are frequently used for lung isolation and one lung ventilation in thoracic surgery and other specialized clinical scenarios. Modern DLTs are large and rigid, and account for half of all tracheobronchial injuries. Their 70 year old design has numerous flaws which limit their safety and clinical utility. Our research team set out to design a new and improved DLT to mitigate these shortcomings, and then test the proposed device to ensure proper function.

**Methods:**

Using published airway anatomy data and computed tomography imaging from 195 thoracic surgery patients, we designed a new DLT with a single size/configuration that would fit into adult surgery patients. This single “Universal design” was intended to replace both left and right sided 35Fr-41Fr DLTs (8 total products), while remaining small in diameter (35Fr). Other design goals included: 1) making intubation easier and safer, 2) allowing full sized therapeutic bronchoscopes to fit into this tube, 3) making the DLT more resistant to dislodgement. After design process completion the proposed dimensions were tested against 195 patients' left and right mainstem bronchi for radiographic fit. Once production prototypes were manufactured, they were tested in large adult Yorkshire pigs and fresh human cadavers for anatomic fit and performance.

**Results:**

The proposed design passed the radiographic fit test in all 195 patients for both left and right mainstem endobronchial placement. Intubation was successful and deemed atraumatic in all pigs and cadavers, and the device appropriately fit in both the right and left mainstem bronchi. Lung isolation was successfully achieved and the device proved resistant to axial force dislodgement.

**Conclusion:**

We propose a new design for a novel DLT meant to replace 8 currently supplied adult configurations with a single, one size/configuration fits all product that allows for large bore bronchoscopy and resists axial force dislodgement.

## Introduction

Lung isolation and one lung ventilation (OLV) are surgical and anesthesia techniques frequently used in thoracic surgery and other specialized clinical scenarios. Thoracic surgeons in the United States perform approximately 530,000 operations per year to address diseases of the lungs, trachea, esophagus, chest wall, mediastinum, and diaphragm, and many of these require lung isolation and OLV (1). There are approximately 4,000 cardiothoracic surgeons in the United States who also perform minimally invasive heart surgery that utilizes similar surgical exposure techniques (2). OLV is a critical step in creating suitable conditions for thoracic surgery (3). This technique enables one lung to ventilate and oxygenate the patient while the other lung is collapsed and immobilized, facilitating surgery of the chest and/or its contents. Surgery on the lung itself requires the organ to be non-ventilated in most cases, especially in minimally invasive and robotic surgery (4–9). Furthermore, other operations in the chest including heart, esophagus, and spine procedures require lung collapse to enable surgical exposure and proper visualization of those structures (10, 11). Finally, OLV provides containment of unilateral pulmonary bleeding or infection, management of bronchopleural fistula or other unilateral pulmonary air leaks, and differential lung ventilation in the critical care setting.

Current routine practice techniques to achieve one lung ventilation include utilization of a bronchial blocker via a single lumen tube or a double lumen endobronchial tube (DLT). OLV is most commonly achieved by use of the DLT, however, due to their shape and size, they can be difficult to pass through the larynx, and are more likely to cause oropharyngeal or tracheal injury than a single lumen tube (12). Their narrow lumens prevent the effective use of diagnostic or interventional bronchoscopes, thus severely limiting tracheobronchial lavage and the delivery of therapies (4). Currently available large and rigid polyvinyl chloride (PVC) DLTs account for half of all iatrogenic tracheobronchial injuries, which can be life threatening (13). Lack of stability within the airway and frequent dislodgement during positioning and surgery disrupts effective OLV, causing prolongation of surgery and possibly even life threatening hypoxemia (14, 15). Lack of a *universal design* also contributes to inefficiencies, with hospitals required to stock eight different sizes and configurations (16–22). Taken together, these deficiencies in the current clinical practice have created a critical unmet need for significant innovations in the design, functionality, and effectiveness of DLTs.

Modern disposable plastic DLTs are modifications of the original Robert-Shaw tube introduced more than sixty years ago (10, 11, 23, 24). These endotracheal tubes contain two separate lumens, one for each lung, and ventilation is separated with the use of endotracheal and endobronchial balloon cuffs. The currently used DLT design suffers from several major drawbacks that negatively affect clinical care: (1) DLTs are large, extremely rigid and bulky. (2) There are two configurations required for placement in each bronchus (left vs. right), and four adult sizes. (3) They contain relatively small sized ventilation passages. (4) The endobronchial balloon cuffs are elliptical in shape and smooth surfaced, which contribute to frequent device dislodgement and subsequent malpositioning. (5) Lung isolation requires separate clamps that can precipitate device dislodgement.

**Figure 1:**
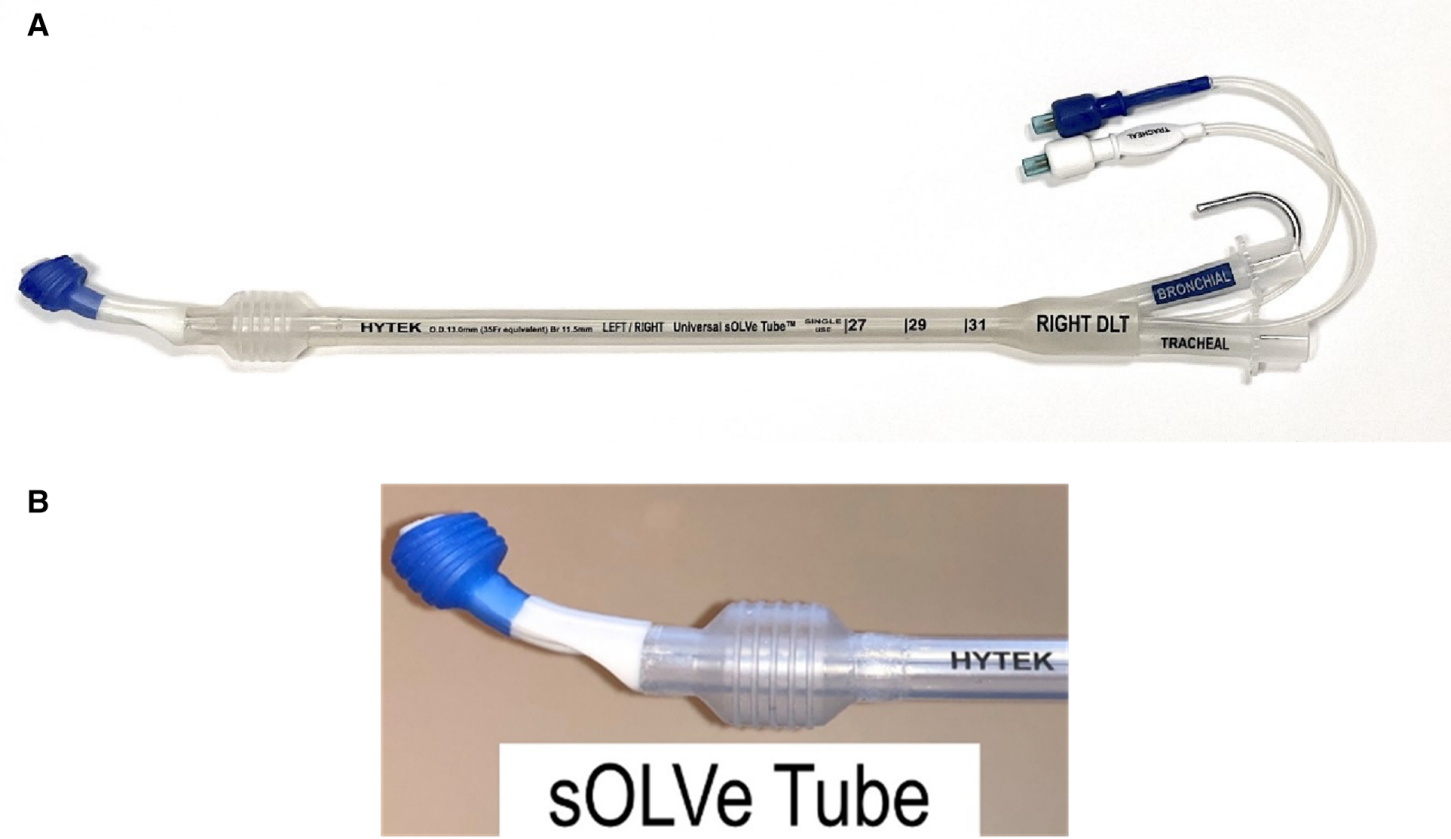
(**A**) sOLVe Tube. (**B**) Distal portion of sOLVe Tube. The cylindrical cuffs with treads for increased grip and seal.

In an effort to address the major drawbacks of the currently available DLTs and to broaden usage in both operating rooms and intensive care units (ICUs), a physician-led team designed and developed the sOLVe Tube (*S*imple *O*ne *L*ung *V*entilation for *E*veryone—Figure 1), a single use, novel, double lumen endobronchial tube. To facilitate ease of adoption amongst current and future practitioners, the sOLVe Tube design was integrated into the familiar DLT blueprint. The device allows for a single DLT configuration to be used for isolation of either the left or right lung.

The sOLVe Tube introduces three major design innovations: (1) universal “one size and configuration fits all” design that enables safe and easy insertion and positioning. (2) flexible lumens that allow for insertion of larger catheters and bronchoscopes, (3) increased stability within the tracheobronchial tree to prevent dislodgement. A primary feature distinguishing the sOLVe Tube from currently available DLTs is that a single design allows the tube to be placed easily and atraumatically in either the left mainstem bronchus (LMB) or right mainstem bronchus (RMB) of most adult patients with an appropriate fit. This tube design replaces the 8 DLT sizes/configurations (35Fr, 37Fr, 39Fr, 41Fr left and right DLT) currently needed to care for adult patients with a single product.

**Figure 2:**
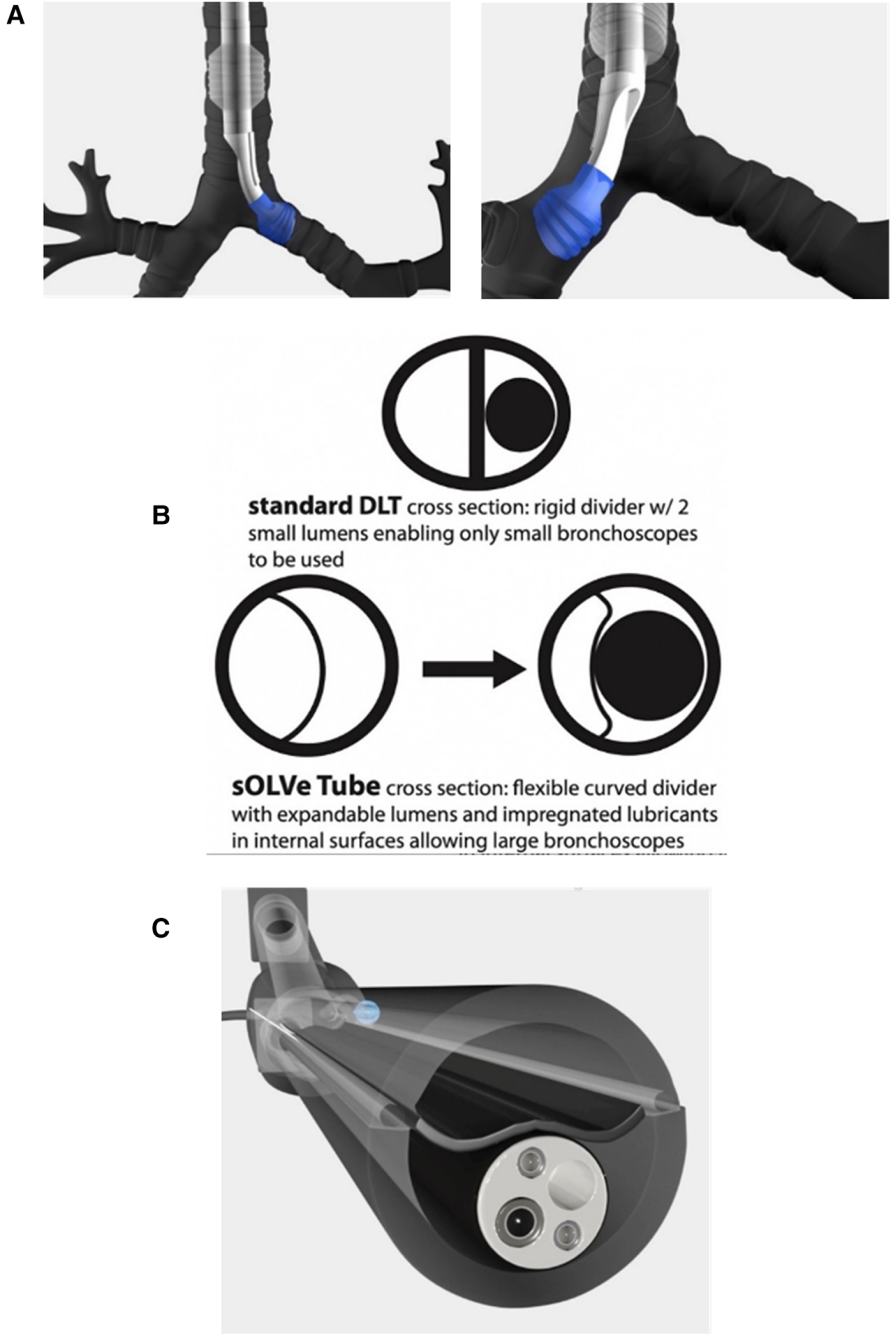
(**A**) Computer animation of sOLVe Tube correctly positioned in the left and right mainstem bronchus with balloon cuffs inflated. (**B**) Cross section of shafts comparing standard double lumen tube versus sOLVe Tube. (**C**) sOLVe Tube with bronchoscope within the shaft. The large bronchoscope deflects the membrane on insertion.

To achieve the universal design goals, new materials and technologies not previously found in DLTs had to be identified and incorporated. Whereas current standard DLTs use rigid polyvinyl chloride (PVC), the sOLVe Tube is constructed from 100% medical grade silicone rubber. The silicone material is much softer and more flexible than most DLTs. This allows the shaft to circumnavigate the individual patient's airway as it maneuvers into the trachea and bronchus, significantly simplifying insertion and increasing safety. The sOLVe Tube is designed in a non-enantiomeric configuration, enabling 180 degrees of rotation within the airway to alternate between left and right mainstem bronchus positioning (Figure 2).

Another major design goal was engineering a tube whose outer diameter was equivalent to the smallest currently available adult DLT (35 French), but that would enable insertion of large bronchoscopes that even the biggest adult DLT (41 French) could not accommodate. This design feature would allow easier and less traumatic endotracheal intubation while also facilitating insertion of large bronchoscope into the airway. To meet this goal the standard DLT shaft layout (elliptical cross section with two small and opposing D-Shaped lumens) was replaced with an innovative design. This new shaft had a round cross-sectional area bisected into two lumens by a thin flexible membrane (Figure 2B). These lumens have equal cross-sectional areas, but different cross-sectional shapes due to the curved and offset nature of the flexible divider. The silicone material enables the dividing membranes to flex and accommodate large bronchoscopes that cannot fit into standard DLTs (Figure 2C).

To facilitate insertion and passage of large bronchoscopes into these expandable lumens, the internal channels of the sOLVe Tube are coated with a water activated bio-lubricant that reduces friction between the scope and tube. This advanced coating, a first for endobronchial tubes, also resists blood coagulation and bacterial colonization, important properties for surgery, trauma, and critical care settings. The sOLVe Tube's pre-coated internal lubricant is long acting and evenly distributed throughout the internal surfaces, thus enabling large endoscopes to pass with ease while also reducing entrapment of instruments in the lumen.

**Figure 3:**
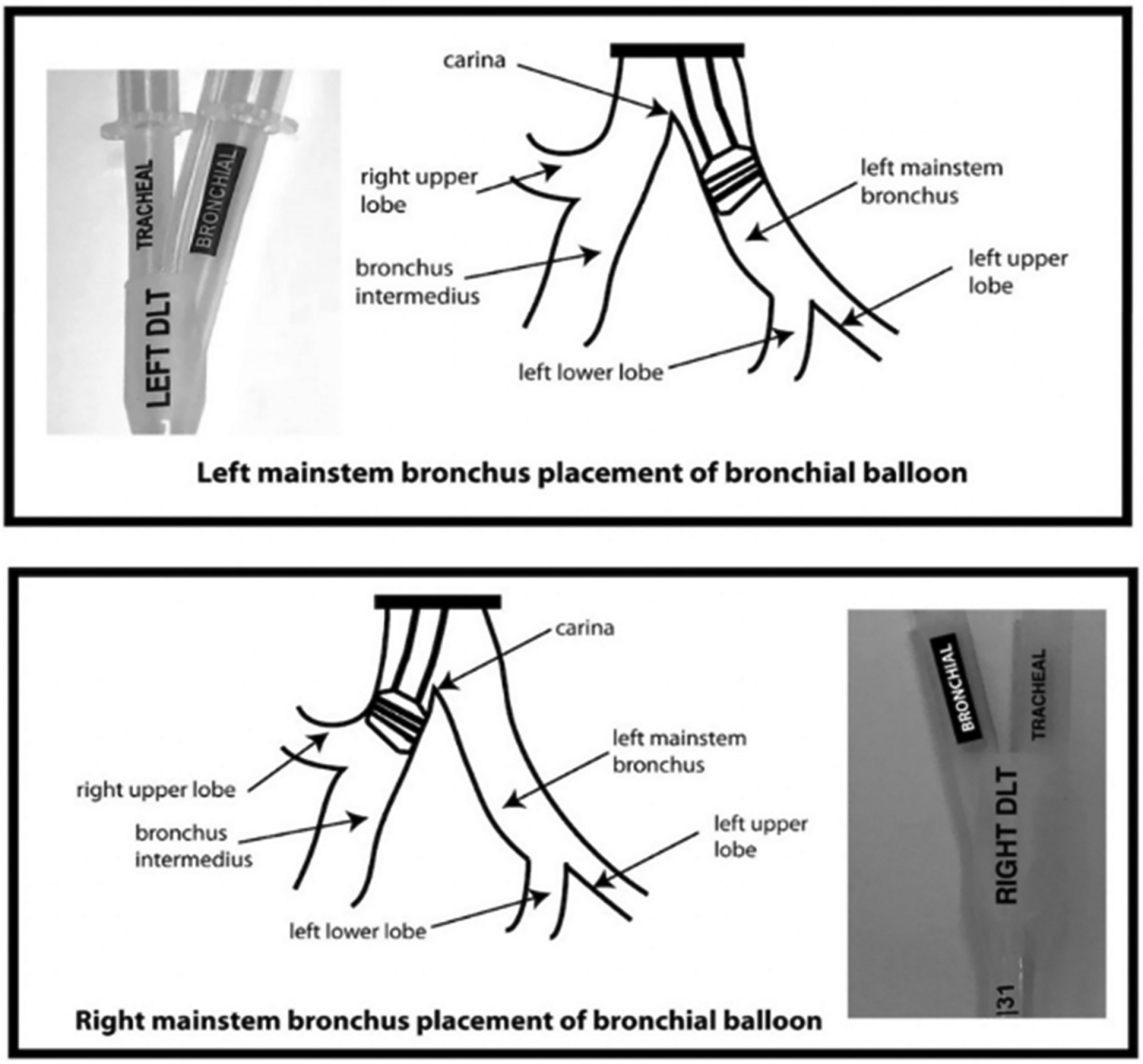
Bilateral Placement of sOLVe Tube—universal design allows for placement of one configuration of the sOLVe Tube in both the right and left mainstem brunchus. (**A**) sOLVe Tube placement in the left mainstem bronchus. (**B**) sOLVe Tube located in the right mainstem bronchus.

Another important design element of the sOLVe Tube is its asymmetrical (trapezoidal) and cylindrical distal balloon with raised ridges that improve the seal between the device and the airway while also reducing device dislodgement (Figure 1B). This feature improves the circumferential air seal while also increasing friction and thus grip by a factor of 27× without changing the pressure inside the balloon. It enables stable positioning above the bifurcation of the right upper lobe and bronchus intermedius (Figure 3). All other currently available right sided double lumen tubes require alignment of a side orifice with the right upper lobe take-off. The sOLVe Tube can be inserted into the right mainstem bronchus and used as a R-sided DLT without the need to first align a side orifice. Alternatively, it can be rotated 180 degrees and inserted into the left mainstem bronchus and utilized as a left-sided DLT (Figure 3).

**Figure 4:**
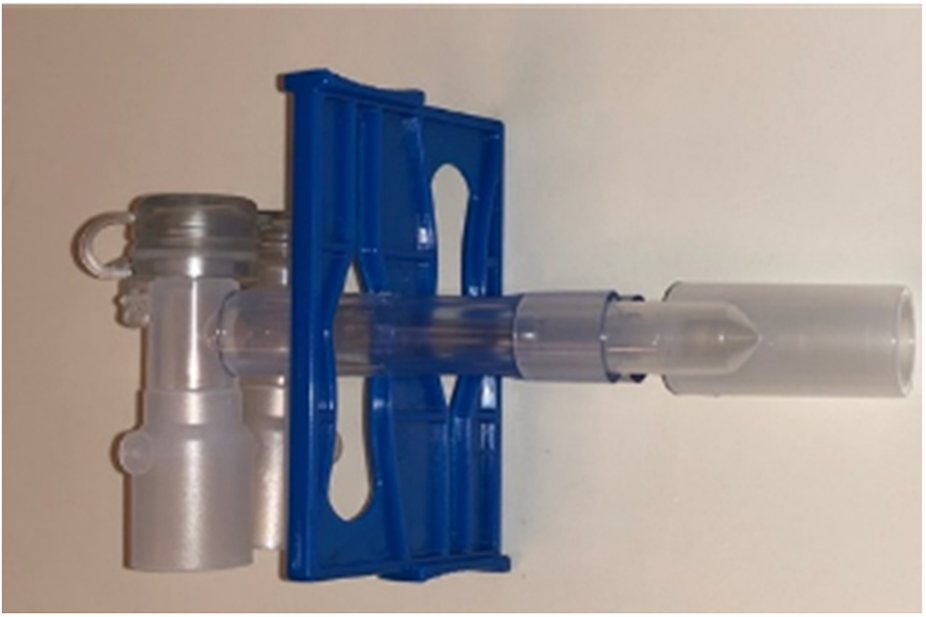
Integrated dual clamp for initiation of OLV.

The ergonomic safety double-clamp, another unique design feature incorporated into the sOLVe Tube, allows for safe and easy occlusion of one limb of the DLT while making it impossible to accidentally clamp both. This integrated patented clamp is light and cannot torque the tube or dislodge it from the airway (Figure 4).

These combined features allow easy and atraumatic endotracheal intubation, and successful endobronchial positioning into either the left or right mainstem bronchus. Our team worked over several years to develop and refine the device using CT-scan guided simulation, large animal testing, and human cadaveric testing.

**Figure 5:**
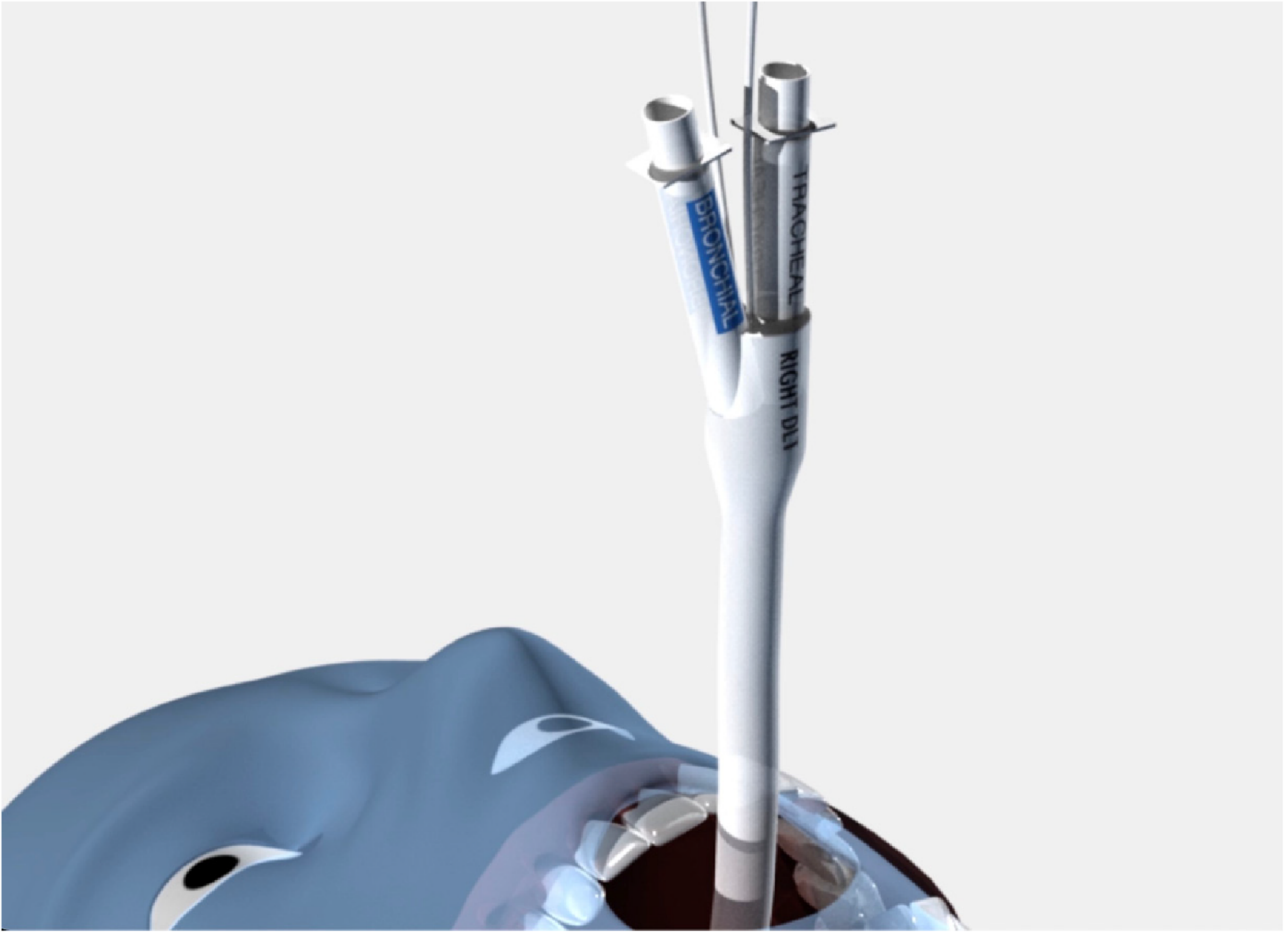
sOLVe Tube positioned in right mainstem bronchus orientation.

**Figure 6:**
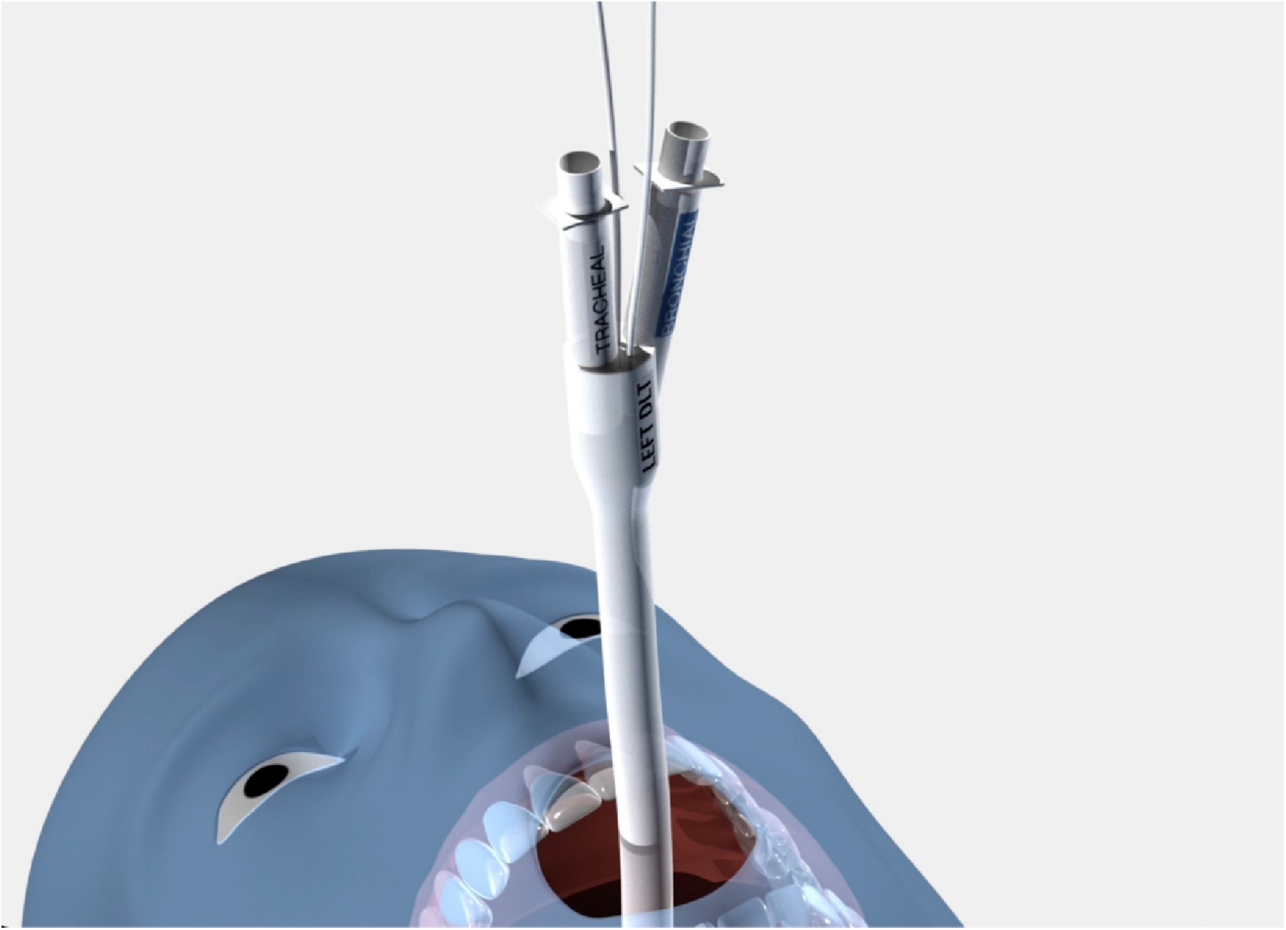
sOLVe Tube positioned in left mainstem bronchus orientation.

**Figure 7:**
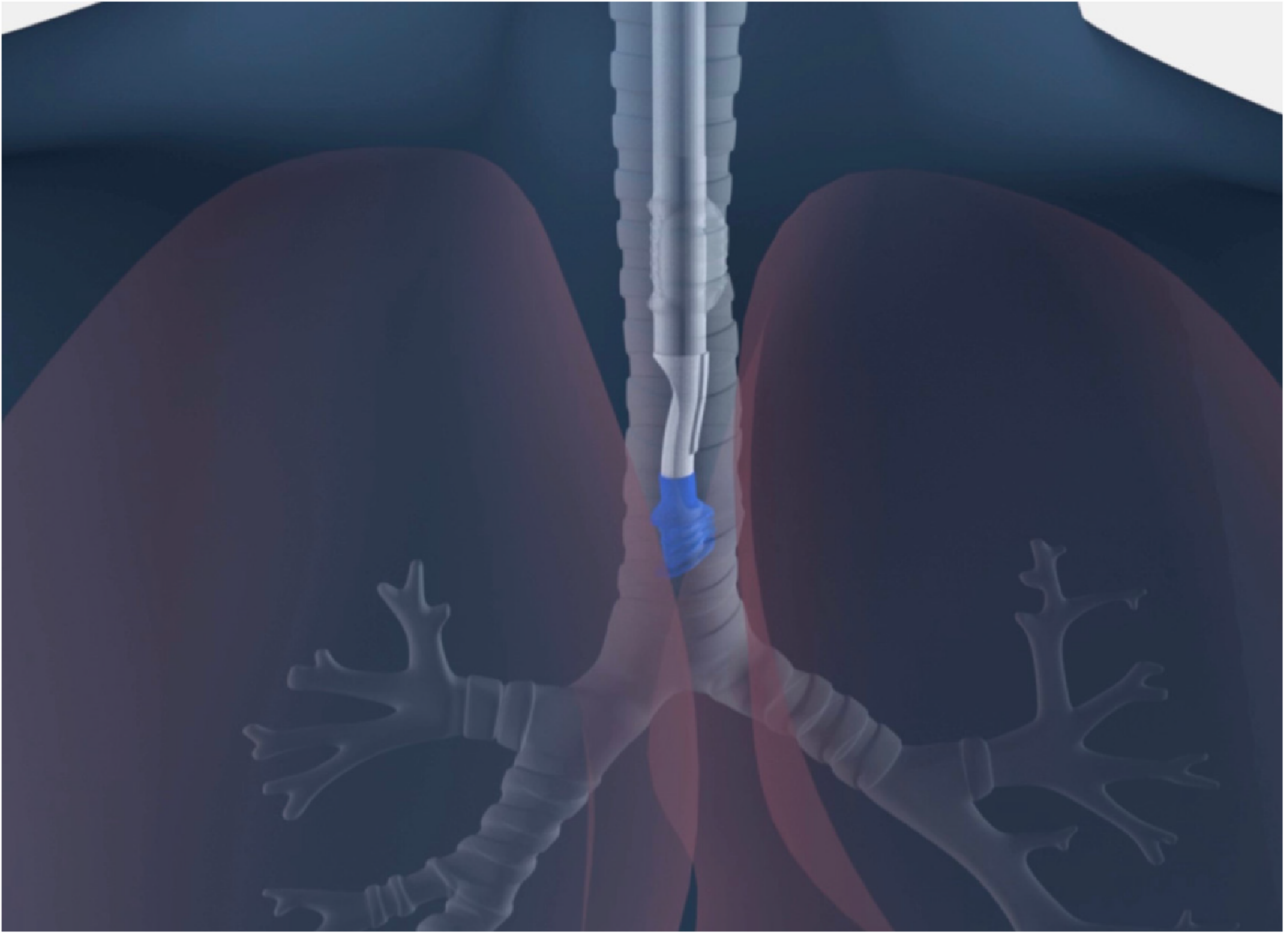
sOLVe Tube located in the trachea.

Figures 5–7 demonstrates the simplified placement technique of the sOLVe Tube. The final location in the right and left mainstem can be seen in Figure 2A.

## Methods

### CT anatomy simulation study

The sOLVe Tube introduces bronchial tip and balloon cuff enhancements that allow for universal design. To determine the correct dimensions of the cuff in both the inflated and deflated position, the inventors searched current literature to identify the variability in human airway anatomy and reported modifications of the DLT (26–30). Additionally, a study was performed to measure the dimension of the RMB in thoracic surgery patients using CT scans of the chest. Specific critical dimensions were measured and then statistically analyzed to define the anatomical shape of the proximal right and left mainstem bronchi and aid in the invention of a truly universal design. This allowed for development of a distal bronchial tip and balloon cuff that would fit properly in both the proximal left and right mainstem bronchi (Figure 1B).

**Figure 8:**
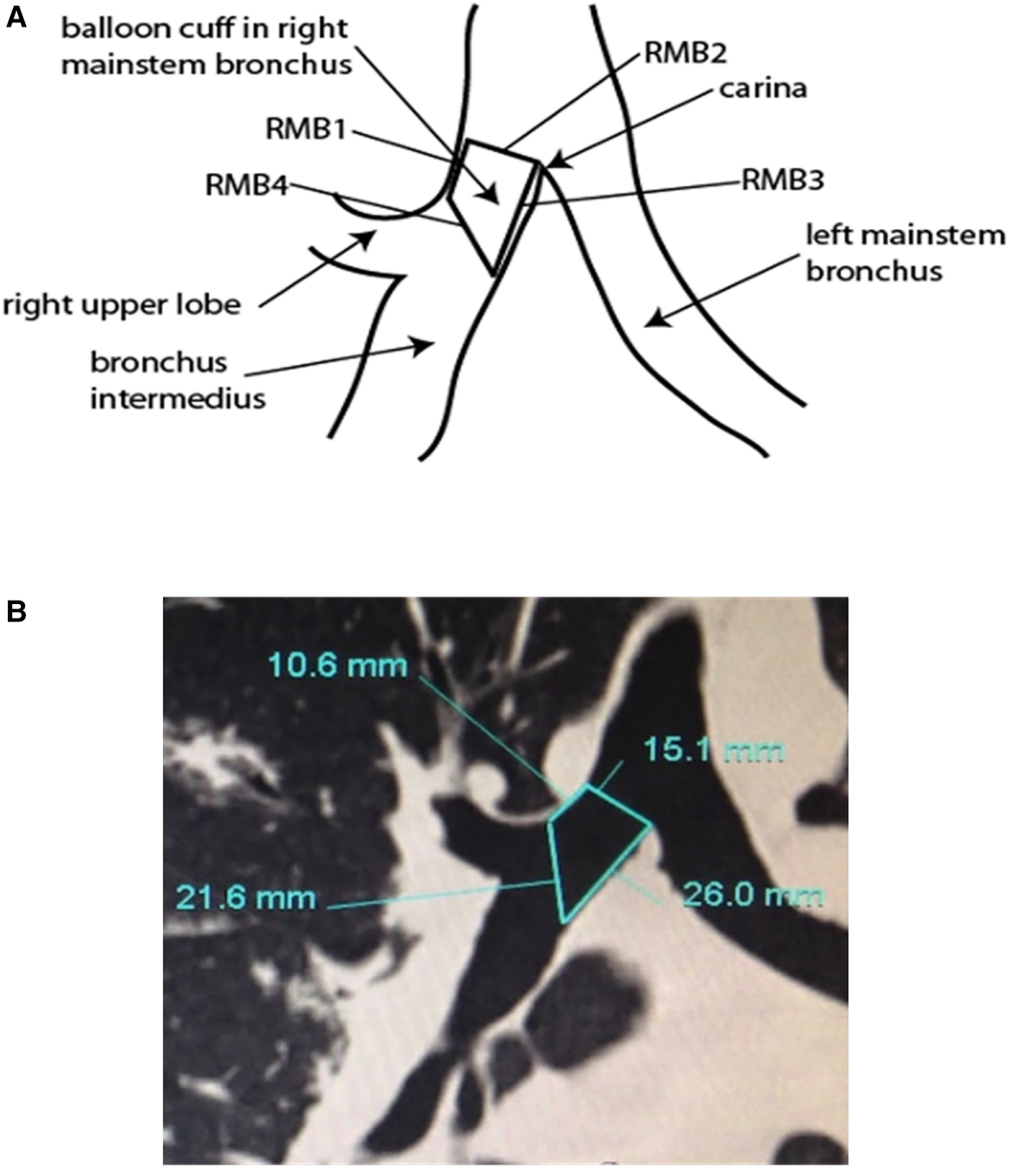
(**A**) Anatomical research for measurement of right mainstem bronchus (RMB). (**B**) Computer tomography of carina including right mainstem bronchus measurements.

Our research team collected airway anatomy data from 195 thoracic surgery patients. In each CT cut, the following lines were drawn and dimensions measured: (1) RMB1, (2) RMB2, (3) RMB3, (4) RMB4 (Figure 8A). RMB1 is the length of the RMB on the right upper lobe (RUL) side, defined as the distance from the RUL takeoff to the perpendicular line drawn from the carina to the RMB lateral wall. RMB2 is the diameter of the RMB from the tip of the carina drawn perpendicularly to the opposing wall of the RMB. RMB3 is the length of the RMB on the carina side. RMB4 is the diagonal line that connects the distal ends of RMB1 and RMB3 together. All dimensions were measured in millimeters (Figure 8B). The dimensions were drawn as closely as possible to the anatomy, with the limitation that the anatomy was not always amenable to straight lines given variability. The data was then analyzed, calculating the mean, median, and standard deviation of each measurement as well as their maximal and minimal values in order to evaluate the variability between patients, focusing specifically on the length of the RMB (RMB1).

The goal of the CT study was to use the anatomical data to design and model a shape for the bronchial balloon that would be a best fit into the highest number of patients, based on the criteria for fit listed below:

### Left mainstem bronchus (LMB) and right mainstem bronchus (RMB) positioning

(1) Ideal position
  (a) The distal end of the balloon is at least 2.5 mm above the secondary carina (dividing the left upper and left lower lobes on the left and the right upper lobe and bronchus intermedius on the right) such that air can easily flow into all lobes.
  (b) The proximal end of the balloon protrudes no more than 1.0 mm above the main carina, such that the rim of the balloon would be visible via bronchoscopy but at least 90% of the balloon's length is within the respective mainstem bronchus
(2) Good position
  (a) The distal end of the balloon is at least 2.5 mm above the secondary carina such that air can easily flow into all lobes.
  (b) The proximal end of the balloon protrudes no more than 2.0 mm above the main carina, such that the rim of the balloon would be visible via bronchoscopy but at least 80% of the balloon's length is within the respective mainstem bronchus
(3) Acceptable position
  (a) The distal end of the balloon is at least 2.5 mm above the secondary carina such that air can easily flow all both lobes.
  (b) The proximal end of the balloon protrudes no more than 4.0 mm above the main carina, such that the rim of the balloon would be visible via bronchoscopy but at least 60% of the balloon's length is within the respective mainstem bronchus.
(4) Unacceptable position
  (a) With the distal end of the balloon at least 2.5 mm above the secondary carina, the proximal end of the balloon protrudes more than 4.0 mm above the main carina such that less than 60% of the balloon's length is within the respective mainstem bronchus.
  (b) If the balloon is advanced to accommodate at least 60% of its length into the LMB or RMB, the distal end moves too close (<2.5 mm) to or even beyond the secondary carina, such that ventilation of all lobes cannot be ensured.

Based on the collected CT data, we hypothesized that the bronchial balloon cuff should have the following dimensions to maximize fit in most patients:

(1) Side 1: 10 mm length
(2) Side 2: Would inflate to occupy a maximum diameter of 24 mm
(3) Side 3: 15 mm length
(4) Side 4: beveled edge that connects side one and side 3.

### Device clinical testing

The sOLVe Tube was tested in both large animals and fresh (non-embalmed) human cadavers to demonstrate appropriate function. The data obtained from the literature and gleaned from the radiology-based clinical trial was used to design the universal distal tip and balloon cuff. Over the years, more than a dozen such designs were prototyped and tested on the bench and in large animals (Yorkshire pigs), with the goal of maximizing the performance of balloon inflation and deflation, symmetrical expansion, and resistance to herniation and/or puncture. The animal studies were conducted at the UCLA Center for Health Sciences, and the cadaver studies were conducted at the UCLA David Geffen School of Medicine Cadaver Processing Lab.

### Animal study

To achieve adequate performance testing, the sOLVe Tube was used to intubate and ventilate large animals during a cardiovascular study requiring an extended intubation period of >6 h. The animals were prepared for the study in accordance with the National Institute of Health (NIH) Guide for the Care and Use of Laboratory Animals. The experiments were performed on two 11 week old female Yorkshire pigs weighing 37 kg and 50 kg. One animal underwent long-term performance testing of the tracheal cuff. The other animal was used to test the long-term performance of the bronchial cuff.

For each experiment, the pig was endotracheally intubated with the sOLVe Tube via direct laryngoscopy, using the included aluminum stylet which was removed after insertion into the trachea. The device was then positioned so that the bronchial balloon was in the left mainstem bronchus, and the time and number of attempts needed to achieve this were recorded. The same procedure was performed for the right bronchus. The device was then attached to a force transducer and the force needed to displace the device 10 mm was measured. The device was then positioned in the correct location for the remainder of the experiment. In one experiment both the tracheal and bronchial balloons were inflated, and the pig ventilated for the duration of the procedure. In the second experiment the device was pulled back and positioned so that the bronchial balloon was above the carina. The purpose here was to simulate positioning of the bronchial balloon in a large human bronchus where maximal inflation would be needed to make a good seal. Next, the ability to successfully achieve lung isolation and OLV utilizing the proprietary dual safety clamp was tested in two methods: (1) with the chest closed by detecting a sharp decrease in pulmonary compliance on initiation of OLV, (2) with the chest open by visualizing each lung inflating independently of the other. Finally, the airway mucosa was inspected via bronchoscopy for any evidence of tissue irritation or injury.

### Cadaver study

We tested advanced, production level prototypes in cadavers to demonstrate the universal design performance of the sOLVe Tube in human beings. Specific performance parameters tested were:

(1) Ease of intubation via direct laryngoscopy (DL) or bronchoscope guidance
(2) Ease of insertion/placement into the left mainstem bronchus (LMB)
(3) Fit/function of the bronchial balloon in the LMB, including sealing of the bronchus
(4) Ease of switching the bronchial tube from the LMB to the right mainstem bronchus (RMB)
(5) Ease of insertion/placement into the RMB
(6) Fit/function of the bronchial balloon in the RMB, including sealing of the bronchus

Once the design and manufacturing process was completed, the final device was tested in fresh (non-embalmed) human cadavers for fit into the tracheobronchial tree. Fresh cadavers were bodies of recently deceased (usually within 24–48 h) donors whose remains were frozen upon receipt but otherwise unaltered so that the tissue anatomy and consistency remained nearly natural. A total of 14 cadavers were used for testing and all were thawed out immediately before use in this trial, ensuring no post-mortem gross anatomic alterations to the tracheobronchial tree. Intubation was performed by anesthesiologists, via direct laryngoscopy first, and if not successful, via bougie or bronchoscope assist. A video bronchoscope was used to photograph and record the results of the testing. Each cadaver was intubated with the styleted sOLVe Tube via direct laryngoscopy (DL), and bronchoscopy assistance (AMBU disposable bronchoscope) was available if needed. The DLT was then inserted into the LMB and the cuffs were inflated. Bronchoscopy was then performed to assess the orientation and position of the distal tip within the LMB. After balloon deflation, the device was pulled back, rotated 180°, and reinserted into the RMB. After cuff inflation, bronchoscopy was performed to assess orientation of the distal end within the RMB. Every step of this protocol was recorded with video and still photos.

## Results

### CT guided simulation study

CT anatomy data was obtained from 195 thoracic surgery patients. One hundred ninety patients (97%) were adults (age 18 or older), and five patients (3%) were children (ages 12, 13, 14, 17, 17). Fifty three percent of the patients were male. Patient cohort demographics are shown in Table 1 and measured RMB anatomy dimensions in Table 2.

**Table 1:** Patient demographics.

	Age	Height (cm)	weight (kg)	PBW (kg)	BMI (kg/m^2^)
Average	60	168	74	62	26
Median	64	168	71	63	25
Min	12	132	40	27	15
Max	89	193	137	87	43
Std. Dev.	18	10	19	11	6

**Table 2:** Patient RMB anatomy dimensions.

*N* = 195	RMB1 (mm)	RMB2 (mm)	RMB3 (mm)	RMB4 (mm)
Average	14.1	13.8	24.1	18.6
Maximum	24.4	21.1	36.1	27.0
Minimum	6.6	6.8	10.4	9.8
STD DEV	3.8	2.3	4.6	3.3
RMB1#<10 mm	26			
RMB1#<9 mm	13			
RMB1#<8 mm	4			
RMB1#<7 mm	1			

The theorized balloon with previously listed dimensions was then two-dimensionally superimposed onto every CT image recorded with the following results:

In the LMB, the proposed distal balloon design with defined dimensional inputs was successfully superimposed into ideal CT-based anatomic position in all 195 patients. In the RMB, the proposed distal balloon was successfully superimposed into ideal position in 182 patients (93%), good position in 9 patients (5%), and acceptable position in 4 patients (2%). There were no patients that were deemed an unacceptable fit.

### Animal study

Intubation via direct laryngoscopy was successful on the first attempt and was defined as easy (airway secured in less than 30 s without assistance of a second clinician). The sOLVe Tube was easily positioned first in the LMB, and then easily rotated and positioned into the RMB (each positioning maneuver took one try and less than 10 s to complete). On the device dislodgement pull test, the sOLVe Tube resisted axial displacement at a force of 0.3 kg, which would easily dislodge a standard PVC DLT. In fact, the device did not move until the force exceeded 0.6 kg, and even then, only 5 mm of displacement was noted. Complete lung isolation was achieved as demonstrated by both closed thoracic measurements of pulmonary compliance as well as open thoracic direct visualization of lung inflation. The dual safety clamp allowed for quick and easy initiation of OLV. Both the tracheal cuff and the bronchial cuffs held a good seal for positive pressure ventilation for the duration of the six-hour procedure without leaking. No evidence of airway endoluminal irritation or injury was noted. Detailed results data is listed in Table 3.

**Table 3:** Summary of large animal (pig) trial results during R&D testing of the sOLVe Tube.

Test Criteria	Result	Pass/Fail
Ease of endotracheal intubation (2 or less attempts in <120 s)	Pig successfully intubated on first attempt within 30 s	PASS
Ease of endobronchial positioning and switching (2 or less attempts in <60 s for each side)	Device positioned into R bronchus in 1 attempt (10 s), device positioned into L bronchus in 1 attempt (9 s)	PASS
Test of lung isolation: (1) closed chest (increase in peak inspiratory pressure >50% when isolating pig lobe), (2) open chest (visualization of single lung inflation)	(1) Closed chest: peak inspiratory pressure increased from 18 cmH2O to 44 cmH2O (144%) (2) open chest: left and right lung individually ventilated per visual inspection	PASS
Test of balloon irritation of airway mucosa (endoluminal camera aided inspection for tears, ulcers, circumferential mucosal sloughing, hematoma)	No tears, ulcers, circumferential mucosal sloughing, hematoma seen	PASS
Balloon cuff performance in a long duration procedure (balloon must hold seal for at least 4 h).	Tracheal and bronchial balloon cuffs held pressure for 6 h without leak	PASS
Balloon cuff “grip” (apply force 0.3 kg, device must not displace more than 10.0 mm)	Force of 0.3 kg did not move balloon. Force increased until 0.68 kg before 5.0 mm movement noted	PASS
Dual clamp isolation performance (clamp can initiate lung isolation in <30 s, both lumens cannot be clamped simultaneously)	Successful lung isolation via clamp achieved in 10 s, both lumens could not be clamped simultaneously	PASS

### Cadaver study

Given the postmortem changes in skeletal muscle (rigor mortis and freezing artifact), many of the cadavers proved to be difficult to intubate given their limited neck mobility and mouth opening. As such, the experiment tested a worst-case scenario for intubation difficulty, which while rarely encountered in clinical practice, poses special challenges when placing DLTs in the airway.

**Figure 9:**
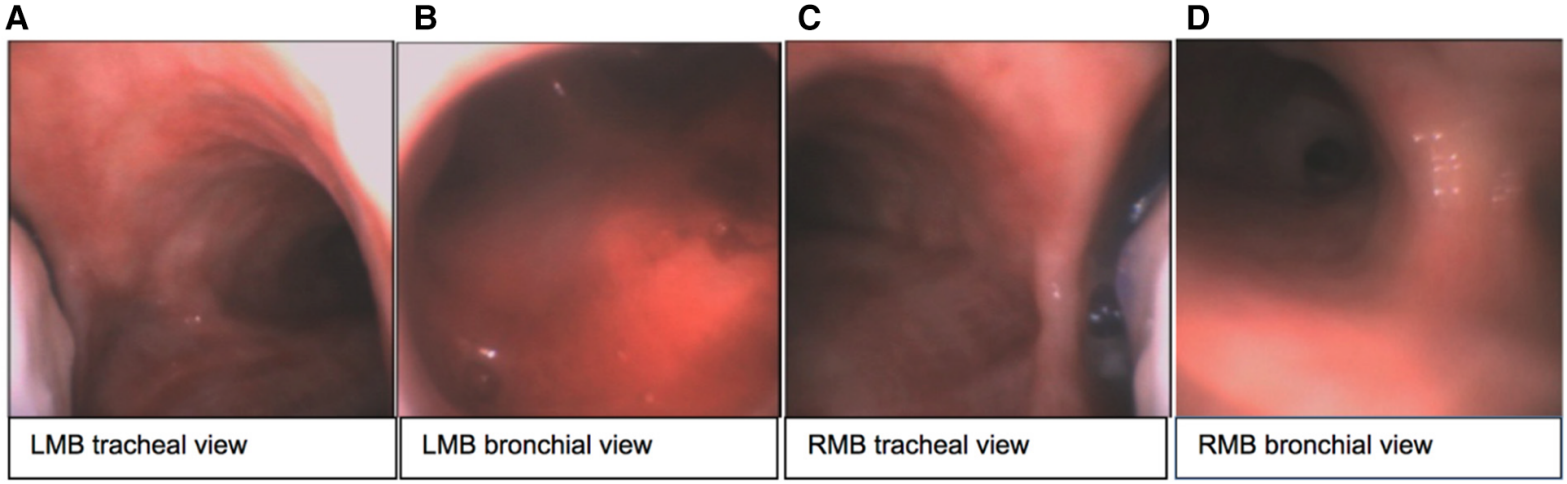
sOLVe Tube positioned in the left and right bronchus of a human fresh cadaver. (**A**) Tracheal view of left mainstem bronchus (LMB) with sOLVe Tube positioned in the left mainstem bronchus. (**B**) View of the distal LMB from the bronchial lumen. (**C**) Tracheal view of right mainstem (RMB) with sOLVe Tube positioned in the RMB. (**D**) View of the distal RMB from the bronchial lumen.

The sOLVe Tube was successfully inserted into every cadaver trachea. Given the severely reduced mouth openings and limited neck mobility of several of the cadavers, the sOLVe Tube's ability to be inserted into these human airways via direct laryngoscopy was especially notable. Furthermore, the soft silicone and flexible design made the bronchoscope assisted intubations effortless. In the opinion of an experienced thoracic anesthesiologist conducting the testing, Standard PVC DLTs would have been exceedingly difficult to insert in these cadavers and would likely result in tissue damage in this subset of difficult intubations encountered in the study. The sOLVe Tube had the proper consistency and curvature at the distal end to easily enter the LMB, and to correctly align with the distal left sided airways (left upper lobe and left lower lobe). The distal balloon cuff, when inflated correctly, totally occluded the LMB without herniating. The device was very effective at lung isolation when positioned in the LMB (L-sided configuration). The sOLVe Tube easily rotated from the LMB into the RMB, and smoothly positioned in the RMB. When the balloon cuff was inflated, the bronchial balloon was correctly positioned within the RMB; the distal tip of the tube remained above the bifurcation of the bronchus intermedius (BI) and right upper lobe, while the proximal end of the balloon did not herniate into the trachea. In no case was the balloon too deep such that it would occlude the RUL take-off. The sOLVe Tube's universal design enabled correct positioning in all cases, including a few which posed clinical challenges due to abnormal anatomy. Its unique design fits into either bronchus and occludes appropriately to facilitate lung isolation (Figure 9).

Table 4 summarizes the testing results for: (1) intubation of the trachea, (2) insertion into LMB, (3) LMB fit, (4) rotation from LMB to RMB position, (5) RMB insertion, (6) fit into RMB. During testing of the fit into the LMB, 12 cadavers were tested. Two were not included (one was an inadvertent protocol breech and the other involved a patient with plastic LMB stent that precluded instrumentation of the LMB. During testing of fit into the RMB, 14 cadavers were tested; twelve (86%) were an ideal fit in the RMB and two (14%) were a good fit.

**Table 4:** Summary of results for cadaver intubation.

Cadaver #	Intubation	LMB insertion	LMB balloon fit	Left to Right flip	RMB insertion	RMB balloon fit
1	Successful, Bronch-assisted	Not attempted^a^	N/A	N/A	Successful	Good fit and alignment of RMB/RUL
2	Successful, bougie assisted	Successful	Good fit and alignment	Successful rotation LMB → RMB	Successful	Good RMB fit, RUL not occluded^b^
3	Successful, Easy DL	Successful	Good fit and alignment	Successful rotation LMB → RMB	Successful	Good fit and alignment of RMB/RUL
4	Successful, Moderate DL	Successful	Good fit and alignment	Successful rotation LMB → RMB	Successful	Good fit and alignment of RMB/RUL
5	Successful, bronch- assisted	Successful	Good fit and alignment	Successful rotation LMB → RMB	Successful	Good fit and alignment of RMB/RUL
6	Successful, bronch- assisted	Successful	Good fit and alignment	Successful rotation LMB → RMB	Successful	Good fit and alignment of RMB/RUL
7	Successful, Moderate DL	Successful	Good fit and alignment	Successful rotation LMB → RMB	Successful	Good fit and alignment of RMB/RUL
8	Successful, Easy DL	Successful	Good fit and alignment	Successful rotation LMB → RMB	Successful	Good fit and alignment of RMB/RUL
9	Successful, Moderate DL	Successful	Good fit and alignment	Successful rotation LMB → RMB	Successful	Good fit and alignment of RMB/RUL
10	Successful, Bronch-assisted	Not attempted^c^	N/A	N/A	Successful	Good fit and alignment of RMB/RUL
11	Successful, Easy DL	Successful	Good fit and alignment	Successful rotation LMB → RMB	Successful	Good fit and alignment of RMB/RUL
12	Successful, moderate DL	Successful	Good fit and alignment	Successful rotation LMB → RMB	Successful	Good fit and alignment of RMB/RUL
13	Successful, Moderate DL	Successful	Good fit and alignment	Successful rotation LMB → RMB	Successful	Good fit and alignment of RMB/RUL
14	Successful, moderate DL	Successful	Good fit and alignment	Successful rotation LMB → RMB	Successful	Good fit and alignment of RMB/RUL

^a^ LMB not intubated due to accidental protocol breech. Technician forgot to perform this first step.

^b^ Tracheal take-off of RUL makes it impossible for any R-sided DLT to occlude and isolate the RUL.

^c^ This cadaver had a large plastic LMB stent, making it impossible to insert any catheter into the LMB.

## Discussion

The device has been deemed substantially equivalent by the FDA and cleared via 510(k) pathway, and as such has met or exceeded the FDA's safety standards for manufacturing, biocompatibility, and clinical use.

**Figure 10:**
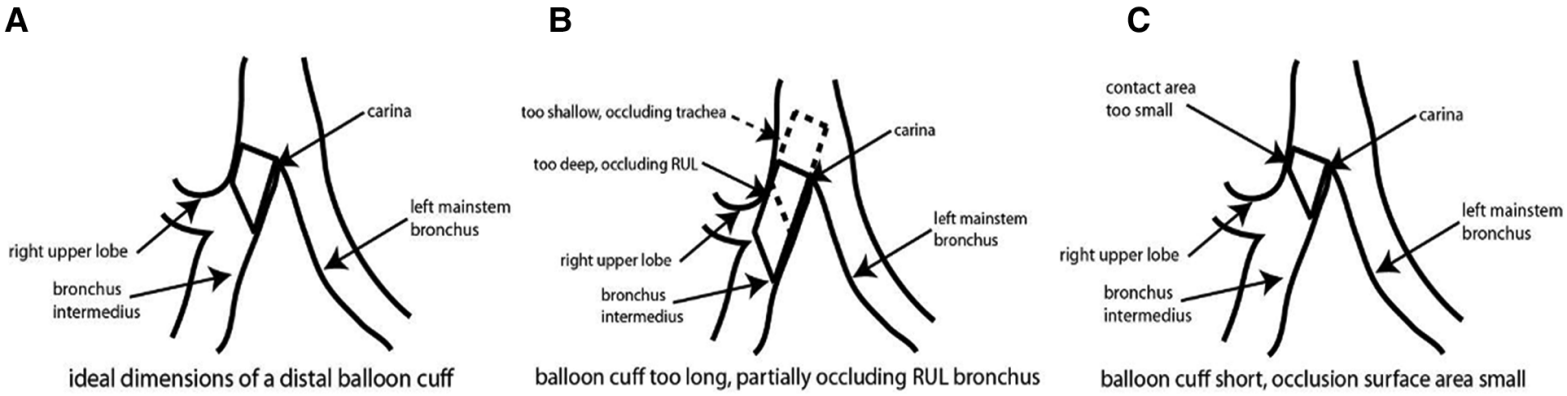
Schematic of sOLVe Tube positioned in the right mainstem bronchus. (**A**) schematic of bronchial balloon in the ideal position within the right mainstem bronchus (RMB). (**B**) Schematic of bronchial balloon that is too long, thus obstructing the right upper lobe take off. (**C**) Schematic of bronchial balloon that is too short for RMB resulting in inadequate contact surface area.

The universal design double lumen sOLVe Tube was conceived after careful consideration of the clinical limitations of the currently available double lumen tubes. Research and development included: (1) evaluation of current literature, (2) analysis of CT imaging of human airway anatomy, (3) bench and ISO testing, and (4) clinical testing in live large animals and human cadavers. By searching the medical literature and then conducting our own anatomic (CT image) study, we identified critical dimensions of the proximal right and left mainstem bronchi, a prerequisite for developing a truly universal DLT (Figure 8). This enabled development of a distal bronchial tip and balloon cuff that would fit properly into both the proximal left and right mainstem bronchi. The functional length of the RMB1 was on average 14.1 mm but ranged between 6.6 mm and 24.4 mm. The short trapezoid dimension of the balloon designed to occlude the RMB needed to fit correctly into this anatomic space. These balloon dimensions needed to properly interface with and seal the RMB. A balloon that was too short could dislodge and leak air more easily because its contact surface area with the airway is minimized. A balloon that was too long could significantly occlude the RUL takeoff or herniate back into the trachea, possibly affecting ventilation of the LMB. The RMB2 dimension (balloon diameter) was less critical because the posterior airway, being membranous, could stretch and flex to accommodate catheters and balloons that appear larger than the dimensions measured in the CT image. Furthermore, the balloon itself could expand to fill a larger bronchus. The ideal balloon cuff would have the longest possible dimensions of RMB1 while still fitting into the majority of patients without obstructing the RUL takeoff (Figure 10).

The animal and cadaver clinical studies confirmed that this design was an ideal fit for both the right and left mainstem bronchi. The fit results of the bronchial balloon in the LMB were not unexpected given that the length of the adult LMB ranges between 3.0–5.0 cm. As such, a balloon with a length of 10 mm on the short side and 15 mm and the long side should easily fit into this space. The fit results of the bronchial balloon in the RMB were very encouraging, considering the challenge of fitting a bronchial balloon above the right upper lobe take-off. The RMB is known to be short in many patients, therefore traditional R-sided DLTs are often challenging to position because the bronchial balloon frequently protrudes beyond the carina when the device's murphy eye is lined up with the RUL orifice. The proposed design, which eliminates the distal end of the tube with the murphy eye replacing it with a natural bevel, combined with the cylindrical trapezoid balloon, matches the natural anatomy of the RMB and thus fits better in this space compared to the current devices on the market. Of note, there was no correlation between the physical dimensions of the patients (height, weight, BMI) and the fit quality of the device in the RMB. All five of the patients who were <18 years of age had an ideal fit with the device. This is because the length of the RMB is not determined by the physical size of the patient, but rather by the physical location where the RUL separates from the RMB.

There are numerous design features that expand the clinical applications of the sOLVe Tube in comparison to current DLTs on the market. First is the innovative shaft design incorporating silicone materials, a flexible semilunar dividing membrane, and an embedded bio-lubricant. Together these three design inputs create a shaft that accommodates passage of large bronchoscopes into the airway (Figure 2). For the first time, therapeutic/interventional bronchoscopes can be inserted into the airway through a DLT, opening the possibility for new therapeutic techniques while also making current procedures safer. For example, lung surgery that requires endobronchial ultrasound (EBUS) can now be conducted with one endotracheal intubation with the sOLVe Tube, which will accommodate the very large EBUS endoscope. With standard DLTs, a single lumen endotracheal tube must first be utilized for the ultrasound, and then a second intubation with a large and rigid DLT is required for the lung resection portion of the case, increasing airway trauma and risk of complications. Procedures like interventional bronchoscopy (used to cauterize bleeding, laser tumors, take biopsies, etc.) which were previously not possible through a DLT due to the large diameter bronchoscopes used, can now be performed without needing to replace the tube. The number of intubations is thus reduced, saving time and money, and lowering complication rates. Massive pulmonary hemorrhage and bronchopleural fistulae, both potentially life-threatening conditions where lung isolation can be lifesaving, can now be effectively managed with simultaneous OLV and interventional treatment, which is not possible with the current clinical practice. The sOLVe Tube's compatibility with therapeutic bronchoscopy and resistance to dislodgement makes it ideal for use in the intensive care unit (ICU). This device opens the possibility of split lung ventilation for respiratory failure, allowing for each lung to receive optimal and possibly different ventilator settings. As we learn more about asymmetrical lung disease in the setting of lung failure, the sOLVe Tube may aid discovery of new ventilation strategies that benefit from precision settings to each individual lung.

A second unique design feature of the sOLVe Tube is its ability to resist device malpositioning or dislodgement. Currently available DLTs are unstable in the airway and easily dislodge with minimal force, and displacement of 10 mm or less can be highly problematic. Such dislodgements can disrupt the surgery given the loss of lung isolation, and in extreme cases, cause severe life-threatening hypoxemia from obstruction of ventilation. In unique cases such as treatment of severe hemoptysis or whole lung alveolar lavage, failure of lung isolation can lead to rapid asphyxia and death. Anesthesiologists are trained to be particularly vigilant with DLT position, which includes frequent position checks with a bronchoscope given that the surgical field is often mobile as the lung is moved and retracted. A recently introduced DLT even embedded a camera into the device for real time position confirmation, but this design increased its already large external diameter while also greatly increasing the cost (25). The sOLVe Tube design tackled this problem via a different approach; rather than visualize and intervene on frequent dislodgements, we set out to reduce and even eliminate them. Airway balloon cuffs are very prone to dislodge because they are spherical, smooth, and rely on pressure to increase contact friction in an otherwise wet and slippery airway (low static coefficient of friction). Our proprietary tracheal and bronchial cuffs are cylindrical and contain circumferential ridges which act as both treads to break up hydroplane and sequential O-rings to seal the airway (Figure 1B). In practice, this design increases the friction and thus grip by a factor of 27× without changing the pressure inside the balloon. Furthermore, the raised ridges function as sequential O-rings that dramatically improve the seal between the device and airway, thus reducing lung isolation failures, especially when there is translational motion (as can happen during surgery or patient transport.

A third major design feature is the sOLVe Tube's universal design with one size fitting all adults. One device replaces all eight DLT sizes and configurations (35Fr L, 35Fr R, 37Fr L, 37Fr R, 39Fr L, 39Fr R, 41Fr L, 41 Fr R). Hospitals would no longer need to discard rarely used DLTs (uncommonly used sizes and configurations) held in inventory that are likely to expire on the shelf. Inventory management would be significantly simplified, especially important for more remote facilities and field hospitals with supply chain challenges. Reduced complexity combined with improved physical properties of this unique device make it easier and safer to use.

The sOLVe Tube opens the gateway for medical treatment of conditions that are not easily treated today, such as massive hemoptysis, bleeding penetrating lung injury, large bronchopleural fistula, and asymmetrical lung infection/pathology requiring prolonged mechanical ventilation in the ICU. Military field medicine could also greatly benefit from adoption of this device. Management of all these conditions currently suffers from a lack of OLV that is compatible with interventional treatment. The sOLVe Tube enables new management that can improve success and reduce overall morbidity and cost. As minimally invasive and robotic assisted surgical, cardiology, and pulmonary procedures replace previously morbid open procedures, successful lung isolation and OLV will become a growing priority. ICU/critical care, especially the management of respiratory failure with complex and asymmetrical lung pathology, will benefit greatly from a DLT that is fully ICU compatible. Positioning the sOLVe Tube as the model device in lung isolation and OLV will improve the quality and cost of medical care.

Extensive research went into the development and execution of the sOLVe Tube. It has been deemed substantially equivalent by the FDA and cleared via 510(k) pathway, as such has met or exceeded the FDA's safety standards for manufacturing, biocompatibility, and clinical use. The universal design was thoroughly tested in radiographic CT-based simulation testing as well as in large animals and human cadavers. The results demonstrate that the device satisfies all the design inputs for a universal endobronchial tube. This device will simplify implementation of lung isolation among anesthesia providers as well as non-operating room locations such as emergency departments and intensive care units. There is a plan to perform size comparison trails for each of the existing sizes of the traditional DLT directly against the one size fits all sOLVe Tube. This randomized clinical trial has been designed and we are currently awaiting funding. Future studies of the sOLVe Tube will include an *in vivo* comparative analysis of the current standard DLT with that of the sOLVe Tube to further highlight the universal applicability of the tube and overall improvements in design over the traditional muti-sized DLT.

## Data Availability

The original contributions presented in the study are included in the article/Supplementary Material, further inquiries can be directed to the corresponding author.
